# Richmond Academy of Medicine and Surgery

**Published:** 1902-03

**Authors:** 


### SOCIETY REPORTS.

### RICHMOND ACADEMY OF MEDICINE AND SURGERY.

Regular meeting; Stuart McGuire, M.D., president, in the chair. Mark W. Peyser, M.D., reporter.

Dr. E. Guy Hopkins read a paper on "Practical Results from Examination of the Stomach Contents." (See p. 801.)

DISCUSSION.

Dr. Wm. S. Gordon said that most of the cases as described by Dr. Hopkins, went from one physician to the other, giving the credit of cure to the last. The doctor had well said that it was just as unscientific to arrive at a diagnosis of Bright's disease without urinalysis, as of stomach troubles without proper examination of its contents after a test meal. Uufortunately, physicians in active practice could not well make these examinations, though it was their duty to do so. A case of his showed the opposite side of the picture. The patient, a woman, was nervous and suffered from an annoying, burning pain at the epigastrium. There was a little cachexia; and vomiting was present, but the vomitus exhibited no blood or coffee-ground sediment. Two analyses of the stomach contents were made by Dr. Hopkins, revealed almost entire absence of hydrochloric acid, and presence both times, of lactic acid and the Oppler-Boas bacillus. An exploratory incision was advised; but full consent was not given. Hydrochloric acid was administered and resulted in two weeks in the entire cessation of all stomach symptoms. The patient previously had craved acids to such an extent that she used them three times daily; and he had no doubt the practice injured her. As soon as the remedy was given her, the craving ceased. The functions of the stomach, Dr. Gordon said, depended largely upon its nervous supply. Given no extraneous troubles and the nervous system in good order, almost anything could be eaten, but not otherwise.

Dr. Wm. T. Oppenhimer believed that the majority of stomach troubles resulted from overeating. He thought the methods, as applied by Dr. Hopkins, were the correct ones for arriving at the proper plans for treatment; but along with them, food adulteration and the foods that produced certain disorders should be studied connectedly, thus ascertaining cause and effect. Most of the baking powders now on the market were undoubtedly productive of great harm.

Dr. Virginius Harrison asked if examination of the stomach contents revealed the cause of the trouble; or was it but an aid in feeding.

Dr. H. H. Levy: It must be admitted that most valuable information may be derived from careful chemical analysis of the stomach contents; but various considerations render impracticable the submission of many of our dyspeptic cases to the expert. Fortunately, careful physical examination will enable us to recognize such unnatural conditions as dilatation of the stomach or gastroptosis; and careful *inspection* of stomach washings at different intervals, and after meals of different composition, will give us valuable information as to both the muscular power of the organ and the relative digestive capability of its secretion. Often a careful inspection of vomitus, with a knowledge of the time at which the next preceding meal, or meals, had been ingested, informs us plainly as to the functional defects of the stomach. Where the indigestion is acute, we often have the most conclusive proof that the digestive failure is due to what we might term a nervous cause. As applying to the more chronic forms, which we denominate dyspepsia, the point made by Drs. Gordon and Harrison that, doubtless inadequate nerve supply to the secretory apparatus of the stomach is frequently the basis of the trouble, seems well taken. The speaker knew a professional man who suffered, when fully occupied, from distressing dyspeptic symptoms who could digest with comfort only a moderate amount of tender meat taken once a day, and with whom all other kinds of food disagreed; but when this gentleman was away from his work, he could take with enjoyment and no subsequent discomfort, a very varied diet. The explanation was that the demands made upon his nerve force by his heavy work were so great as to leave too small an amount to properly stimulate the secretory glands of the digestive tract.

The success attending the administration of strychnia or nux vomica and of the hypophosphites in cases of atonic dyspepsia was notable. He had most satisfactory results from the employment of tincture of nux vomica, in ten drop doses, mixed in a cupful of hot water, slowly sipped, about a half-hour before meals.

Dr. Levy did not mean to discountenance systematic chemical and microscopical examination of the stomach contents for purposes of diagnosis and determination of proper dietetic and medicinal treatment. In obscure or intractable cases, we should be glad that we may have the assistanee of an expert; and he appreciated the work done by the reader of the paper.

Dr. Gordon said he did not mean to undervalue the influence of either the quantity or quality of food in stomach disorders. Patients did have dyspepsia from overloading the stomach; but examination of its contents revealed what was at fault. Diet had to be corrected in nearly every instance; and nature would then react if the nervous system were in good order and no abnormal extraneous condition existed. In this connection Dr. Hopkins had examined the various text-books and found that a number stated that if the correct amount of hydrochloric acid were secreted, the gastric juice would invariably contain sufficient pepsin. He did not believe this, for the blood might not contain the necessary quantity of albuminous materials for the latter, and yet the required quantity of the former could be present.

Dr. Levy said he did not remember having seen or heard the point raised; yet he believed that pepsin was not always present in the gastric juice in sufficient amount. Mineral acids, as phosphoric, would aid digestion as well as hydrochloric; and if food remained undigested too long after the administration of these acids, it looked as though the quantity of pepsin were not enough. In cancer, in which there was great alteration of structure and absence of hydrochloric acid, it was certainly often observed that food was rejected as much as twenty-four hours after its ingestion.

Dr. Hopkins, in closing the discussion, said that the lactic acid in Dr. Gordon's case was attributed to the bread of the Ewald test meal, but there was none after the Boas meal. Replying to Dr. Harrison's question, he said that the cause was very often found by examination of the stomach contents; for instance, total and persistent absence of free and combined hydrochloric acid indicated permanent atrophy of the gastric glands. In other cases, while the cause might not be well defined, the therapeutic indication of the analysis might be invaluable, as in cares of subacidity where the free acid was deficient or absent, and the combined present: Administration of hydrochloric acid would then stimulate its secretion by the stomach. Regarding the formation of pepsin, Hemmeter, Eichorst and others stated that when free hydrochloric acid was formed, there was always a sufficient amount of pepsin. There were no reports of analyses, the statement being based presumably upon cases in which the amounts were estimated.

